# The hard way from the Beveridge to the Bismarck model of health finance: Expectations and reality in Russia

**DOI:** 10.3389/fpubh.2023.1104209

**Published:** 2023-03-14

**Authors:** Sergey Shishkin, Igor Sheiman

**Affiliations:** ^1^Center for Health Policy, National Research University Higher School of Economics, Moscow, Russia; ^2^Chair of Health Management and Economics, National Research University Higher School of Economics, Moscow, Russia

**Keywords:** health finance, health finance functions, health finance models, mandatory health insurance, collection of funds, pooling funds, purchasing health care, Russia

## Abstract

Most post-Soviet countries have introduced mandatory health insurance (MHI) systems which completely or partially replaced national health systems known as budgetary models. In Russia, an attempt was made to introduce a competitive MHI model with multiple health insurers. The current MHI system has, however, acquired an increasing number of features inherent in the previous budgetary model. This study analyzes the institutional characteristics and the outcomes of a new mixed model. A combination of two analytical approaches is used as follows: (1) considering three functions of the financing system (revenue collection, pooling funds, and purchasing healthcare) and (2) exploring three types of the model regulation (state, societal, and market). We analyze the types of regulation that are used to implement each of the three financial functions. The model has contributed to more sustainable health funding, its geographical equalization, and service delivery restructuring, while the implementation of its purchasing function has many unsolved problems. We highlight the dilemma of the further development of the model by (a) continuing to replace the remaining market and societal regulatory mechanisms with state regulations or (b) developing market mechanisms and thereby strengthening the impact of health insurers on the health system performance. Lessons for countries considering the transformation of their budgetary health finance model to the MHI model are presented.

## 1. Introduction

Most post-Soviet countries have completely or partially replaced their national healthcare finance system, which is often referred to as the Beveridge model or the budgetary model, with mandatory health insurance (MHI)—the Bismarck model—which is a statutory public scheme of healthcare financing based on earmarked contributions of specified actors to stand-alone funds (1). In 1991, Russia was one of the first post-Soviet countries to introduce MHI.

The new finance system raised expectations including the possibility of increasing health funding (which had traditionally been low); promoting provider competition, patient choice, and the cross-border movement of financial resources and patients; and improving the performance of the healthcare system. Competition among insurers was seen as a driving force to protect patients and make more effective use of resources (2).

The Russian MHI model was initially formed under the influence of theories about the design of an effective public health system. Liberal economists and the World Bank were active in calling for a competitive MHI model with consumers' choice of insurers and competition among providers (3). The competitive model, however, faced serious problems due to the lack of market institutions and the short window of opportunity for large-scale reforms. The law on health insurance was adopted in 1991 and the MHI system was introduced in 1993. The desire to not miss the chance to receive a new source of health funding—earmarked for contributions to MHI funds—prompted initiators of the reform to ignore the number of institutions needed to build a competitive MHI model (4). In this difficult socioeconomic transition from a command economy to a market one, a delay in introducing the new model was seen as taking the serious risk of losing MHI contributions as a new source of funding.

In the course of reforms over the next 30 years, the MHI system has evolved substantially, with an increasing number of characteristics inherent in the traditional budgetary model and a diminishing number of characteristics of a market-driven competitive model.

This trend has prompted a number of questions: What kind of healthcare finance system has been built in Russia? What is the outcome? Were the initial expectations met? What are the prospects for the further transformation of the Russian MHI model? What lessons can be learned from the Russian reform in countries considering a transition from the Beveridge to the Bismarck model?

Although these reforms have been addressed in some international studies (5, 6), satisfactory answers to these questions have yet to be found. This study analyzes the institutional characteristics, outcomes, and prospects of the mixed healthcare finance model.

## 2. Methods and data

### 2.1. Study design

We followed a four-step methodological framework. The first stage involved an analysis of the institutional features of the Russian MHI system. The combination of two theoretical and methodological approaches was used. We followed the functional approach to analyze the healthcare finance systems, which was proposed by Kutzin (1) and has been used in many studies and the official documents of the WHO. According to this approach, any healthcare finance system performs the following functions: the collection of financial resources, their pooling, and the purchasing of healthcare. The subjects of the analysis are the institutions that implement these functions.

As an analytical tool for addressing such functions, we used an approach suggested by Rothgang et al. (7). The three types of model regulation are state, societal, and market. The first type is regulation through power coercion; the second is regulation through collective bargaining between public actors who are not authorities; and the third is regulation through market interactions.

The second stage is the evaluation of the MHI model's contribution to the performance of the healthcare system in Russia, including its impact on revenue collection, its allocation across regions and the sectors of service delivery, pooling funds, service delivery restructuring, and the accessibility and quality of medical care. To describe the outcomes, we used qualitative characteristics and quantitative indicators that highlight them to the greatest extent.

The third stage is the identification of unsolved problems of the MHI model. We followed the functional approach with a focus on the purchasing function. We mainly used the qualitative characteristics of the institutions that facilitate (or complicate) these functions.

The fourth stage is the discussion of the current state of the healthcare finance system in Russia and the potential ways it could be transformed in future.

### 2.2. Data sources

We extracted data from national and international databases and reports and calculated secondary estimates. Regulatory documents on health finance and international and Russian literature on the trends in the health system over the last 3 decades were used. We also used the gray literature related to the Russian health finance system, including those in limited circulation, unpublished documents, memorandums, and presentations from our personal collections covering more than 30 years.

This was supplemented with data from our surveys completed by physicians and interviews with senior health managers, including managers of regional MHI funds, conducted over the last decade (the latest was in 2019 before the COVID-19 pandemic).

## 3. Institutional characteristics of the current MHI system

**Figure 1:**
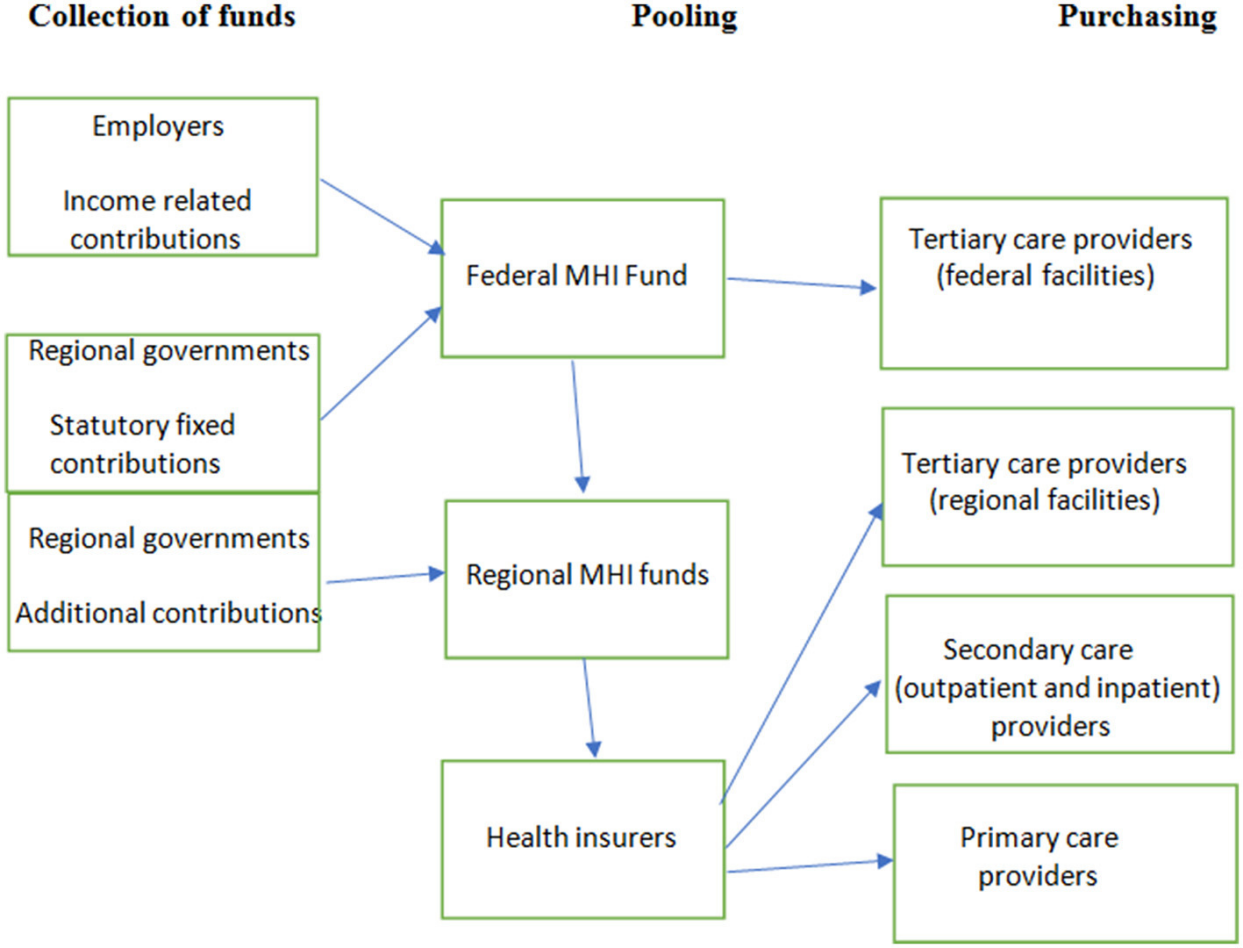
Financial flows in the Russian MHI system.

A scheme of the Russian MHI system is presented in Figure 1.

### 3.1. Revenue collection

The major sources of funds are the mandatory contributions of employers and regional governments to MHI for the working and non-working populations, respectively (employees do not pay directly). The rates of contributions for the non-working population vary according to regional differences in the costs of medical services. Contributions are paid into the federal MHI fund and are then allocated to 86 regional MHI funds that act as operators of the regional healthcare finance systems. The regional government may transfer supplementary contributions to the regional MHI fund for the non-working population, which is a budgetary contribution exceeding the mandatory regional rate of contribution. Thus, the revenue of a regional MHI system consists of federal allocation and supplementary regional contributions.

The earmarked nature of contributions indicates that there is a sustainable flow of funds into the healthcare system. The funds are less dependent on the priorities of budget allocation, which have traditionally been skewed to non-health sectors, particularly defense. However, this dependence has not disappeared completely, partly due to the relatively low rate of the employers' contribution-−5.1% of the payroll—compared with 12–18% in most Central and Eastern European countries (8). In 2020, 41.5% of the federal MHI fund revenue was collected from the general budget revenue, including 31.1% from mandatory regional contributions for the non-working population and 10.1% from the federal budget as budgetary transfers for the compensation of fund shortages to cover the cost of the package of medical benefits (9).

Revenue collection is based on the cost of the annual federal program of state guarantees of free healthcare (the program), which determines the package of medical benefits. This package includes practically all medical services and covers the entire population, although informal rationing is very common in practice. Regions develop their own programs of state guarantees. They have a uniform package of benefits, while its funding is more generous in the richer regions (10).

### 3.2. Pooling funds

MHI contributions are pooled in the federal MHI fund to ensure the equalization of regional funding. The pooled funding is allocated to regional MHI funds according to an age/sex-adjusted capitation rate and the cost of care in different regions. Subsidies from the federal MHI fund to regional MHI funds reduce gaps in healthcare funding across regions but do not result in equal spending per capita throughout the country. The richer regions supplement MHI funding from their general budget sources and spend up to three times more on healthcare than poorer regions. Pooling at the regional level is designed to ensure the risk-adjusted funding of health insurers by regional MHI funds. These insurers are mostly private companies that carry out a number of functions in the MHI system. Currently, there are 29 health insurers. Citizens are entitled to select an insurer. They are funded per enrollee, with risk equalization by regional funds—the redistribution of funds to health insurers who have a relatively high share of risks. The system of risk adjustment is simple, taking into account only the age and sex of the enrollee.

There is limited health insurer competition. Health insurers attract people through customer services (issuing MHI policies more quickly, better processing of patient complaints, and call center quality), but there is no competition on the benefits package, the size of the premium, or the quality of care. These are excluded by the design of the MHI system. Health insurers cannot offer insurance plans with variable premiums or a limited network of providers with specific benefits and premium rates, which is the case in many countries with multiple purchasers of care (e.g., Germany, the Netherlands, Switzerland, and Israel). A declared quality competition (“the best insurers contract the best providers”) is hard to implement due to the lack of consumer information. The decisions to collect such information are not made by the insurers themselves. They work under the pressure of administrative bodies. The results of insurers' “thematic expertise” of provider performance may be useful, but they are not made public and are rarely used by health authorities (11).

There is some element of risk sharing between the regional MHI fund and health insurers, which is also a part of the pooling scheme. In the case of underspending (when an insurer's healthcare spending falls below the insurer's revenue), health insurers must return most of the savings to the fund. The presumption is that MHI financial resources belong to the federal government, except for administration costs (which are specified by the regulation). In the more common case of overspending, health insurers can apply to the regional MHI fund for subsidies that are paid from a so-called “normalized insurance reserve” (5% of regional MHI revenue). It is operated by the regional MHI fund and acts as a pool to ensure the solvency of health insurers (11).

Health insurers are liable for financial risks only within the limits of their capitation-based revenue. The rest of the risks are borne by the regional MHI fund and medical organizations. The proportion of risk-bearing is not determined explicitly.

### 3.3. Purchasing care

Purchasing care in Russia is a combination of centralized planning and the direct contractual interaction of payers and providers. The major instrument of purchasing is planned care utilization across sectors of the healthcare system with further allocation of the planned volumes of care to each service provider. Planning in regions is based on utilization targets (e.g., the number of physician visits, hospital admissions per capita), and unit cost targets (per visit, per admission) established in the program. Over the last 2 decades, the annual programs have been issued by the federal government. While utilization targets are implemented nationwide, adjustment of the targets to regional health needs has been allowed recently (12). National utilization planning promotes service restructuring by ensuring a shift of care from inpatient to outpatient settings, strengthening primary care, and developing daycare centers, among others. Accordingly, federal and regional utilization targets are set and used for contracting payers and providers.

The purchasing function is shared by the commission for the regional program of MHI (the commission) and health insurers. The commission acts as a mix of state and societal regulations. It represents all actors of the healthcare system and acts as the collective purchaser of care. However, the voice of the individual actors varies substantially. The regional health authority and MHI fund play a major role in decision-making about the allocation of volumes of care and funding. Some health insurers are involved in the discussion of plans in the commission, but their role is limited. They contract medical facilities for the provision of care authorized by the collective purchaser.

After planning and negotiating volumes of care, health insurers contract providers for the delivery of care and to pay their bills. The reimbursement is based on provider payment methods used in the region (they determine the units of care that are subjected to reimbursement). The underprovision of the planned volume indicates that a provider will not receive the planned amount of funding, while overprovision might be not reimbursed. The contractual volumes can be adjusted through a new round of negotiations with providers with some chance of setting higher volumes. Sometimes, payments are made only after court proceedings. Thus, some risks are borne by providers.

Provider payment methods are determined by the federal and regional authorities. Uniform payment methods are used in all regions (with minor variations)—capitation for primary care and the diagnosis-related group (DRG) method for inpatient care. Polyclinics as primary care providers are paid additionally by fee-for-service for preventive and some other selected services. Capitation payments can be reduced when a polyclinic has fewer physician visits than the negotiated plan. The DRG-based payment scheme has more than 500 groups. The rates are usually adjusted for hospitals that lose revenue under this method. The so-called “coefficients of DRG” are determined for such hospitals (13).

### 3.4. Governance of MHI

Governance is highly centralized with the federal MHI fund at the top of the system. The design of the financial flows and payment schemes is the joint responsibility of the federal Ministry of Health and the federal MHI fund with the former having a leading role. Although the legislation sets the responsibility of regional MHI funds for the implementation of the MHI scheme in each region, it has to follow federal decisions on most issues of governance and funding, including planning, payment methods, and patterns of resource use. Discretion on decision-making is limited to minor operational areas. Health insurers act as billing companies paying for the volumes of care that are determined by the commission.

MHI funds are managed by a board and an executive director. The board includes representatives of state authorities, health insurers, professional medical associations, and trade unions of health professionals. The board is the institution of societal regulations. However, the attitude of health authorities is usually the most important factor in the decision-making of health policy and on the allocation of resources. According to one of our respondents, “*My attitude when I worked as a director of a health department was very simple. What is an MHI fund? It is a financial division of the department. Nothing more.”*

The design of the MHI system does not treat providers as independent contractors. The managers of state-owned facilities are hired and fired by health authorities. The major decisions on the capacity of such facilities, the scope of services, and their involvement in national and regional vertical programs are made by administrative bodies. There are many other limitations to the operational autonomy of providers as state-owned entities. Thus, the major advantage of contracting—the separation of purchasers and providers (1)—is not fully utilized in the current MHI model.

## 4. Contribution of the MHI model in improving the performance of the healthcare system

### 4.1. Impact on revenue collection

**Figure 2:**
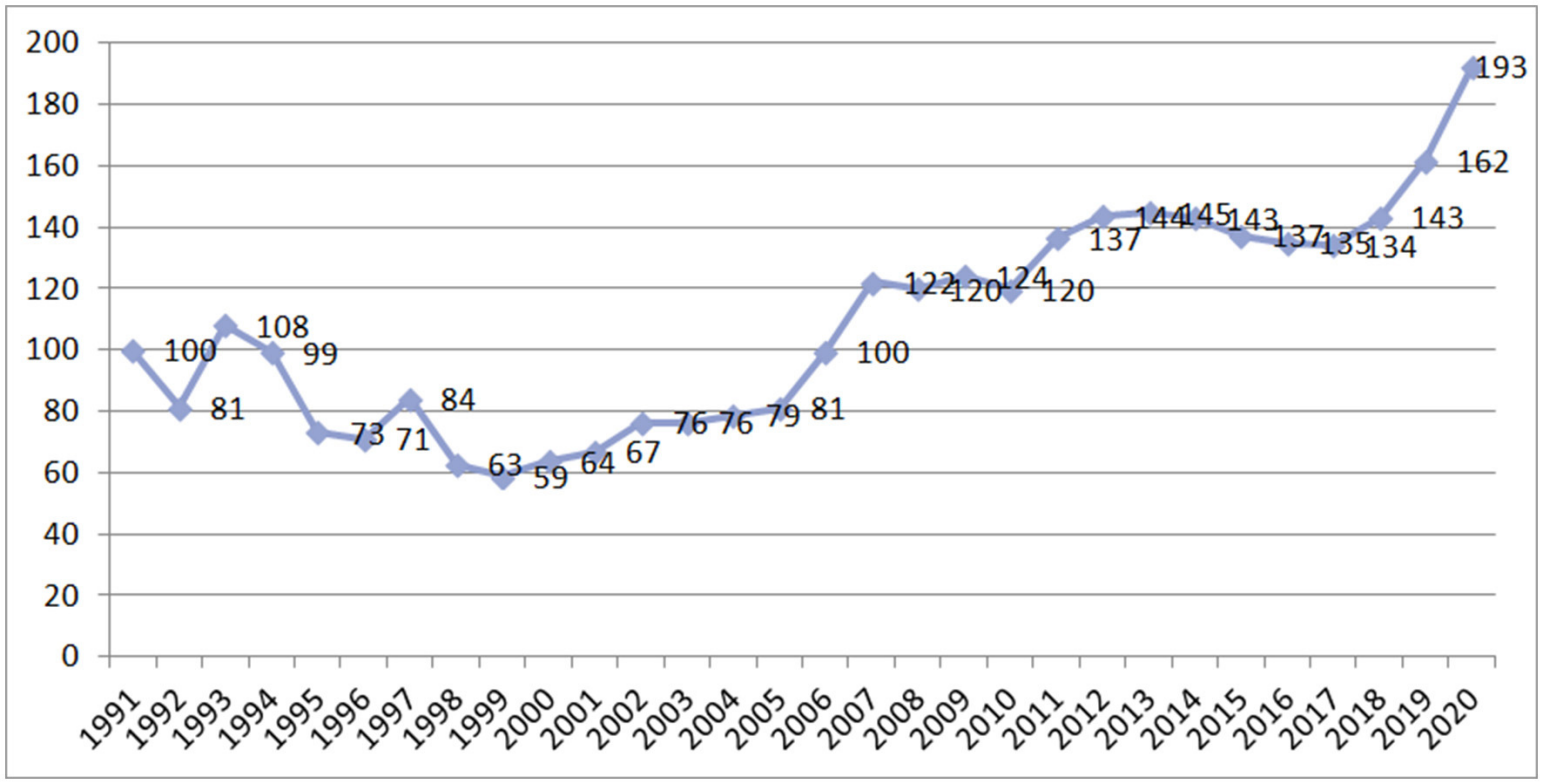
Public funding of health care at constant prices in Russia in 1991–2020. *Source*: Authors' estimates based on official data.

The introduction of the MHI model allowed the earmarking of a substantial portion of healthcare revenue. According to one respondent, the head of the national medical association, “*In the 1990's, there was no alternative, there was no money for healthcare at all, and mandatory health insurance was introduced as an additional tax, which at least somehow supported the system*.” In the 1990's, this earmarking mitigated the negative effect of transformational economic crisis during the shift from a planned to a market economy. The decline in healthcare funding was not as deep as in other industries of the social sector—mostly due to employer contributions as a new source of health funding (14). Public health funding (MHI contributions and budgetary allocations) reduced in real terms until 1999. The 1991 level was reached only in 2006 (Figure 2).

The dependency of MHI revenue on general budget priorities remains high since the contributions for the non-working population are made by regional governments, and the federal government increasingly subsidizes regional MHI schemes (15). The role of budgetary allocations to MHI is increasing, which is the prevailing trend in many OECD countries (16).

### 4.2. Impact on regional equity

The centralization of MHI revenue contributed to the equalization of healthcare funding across regions. The allocation of MHI revenue has been focused on strengthening healthcare funding in poorer regions. In 2010, total public healthcare funding per capita (MHI and budgetary health expenditure) in the richest regions was 3.8 times higher than that in the poorest regions, whereas, by 2018, it was three times higher. Regionally, MHI funds provide some equalization of healthcare funding across local communities through capitation formulas for allocating resources to health insurers. The equalization policy within a budgetary system was much less radical due to the presence of many legislative barriers (17).

Regional equity is also strengthened by cross-boundary flows of patients—mostly from poor to rich regions. The number of patients who received inpatient care outside their region of residence has increased over the last two decades to 16% of the total number of hospital admissions in 2020 (9). This is more evidence of the free movement of money in the Bismarck model compared with the former Beveridge model in Russia.

### 4.3. Impact on service delivery restructuring

**Figure 3:**
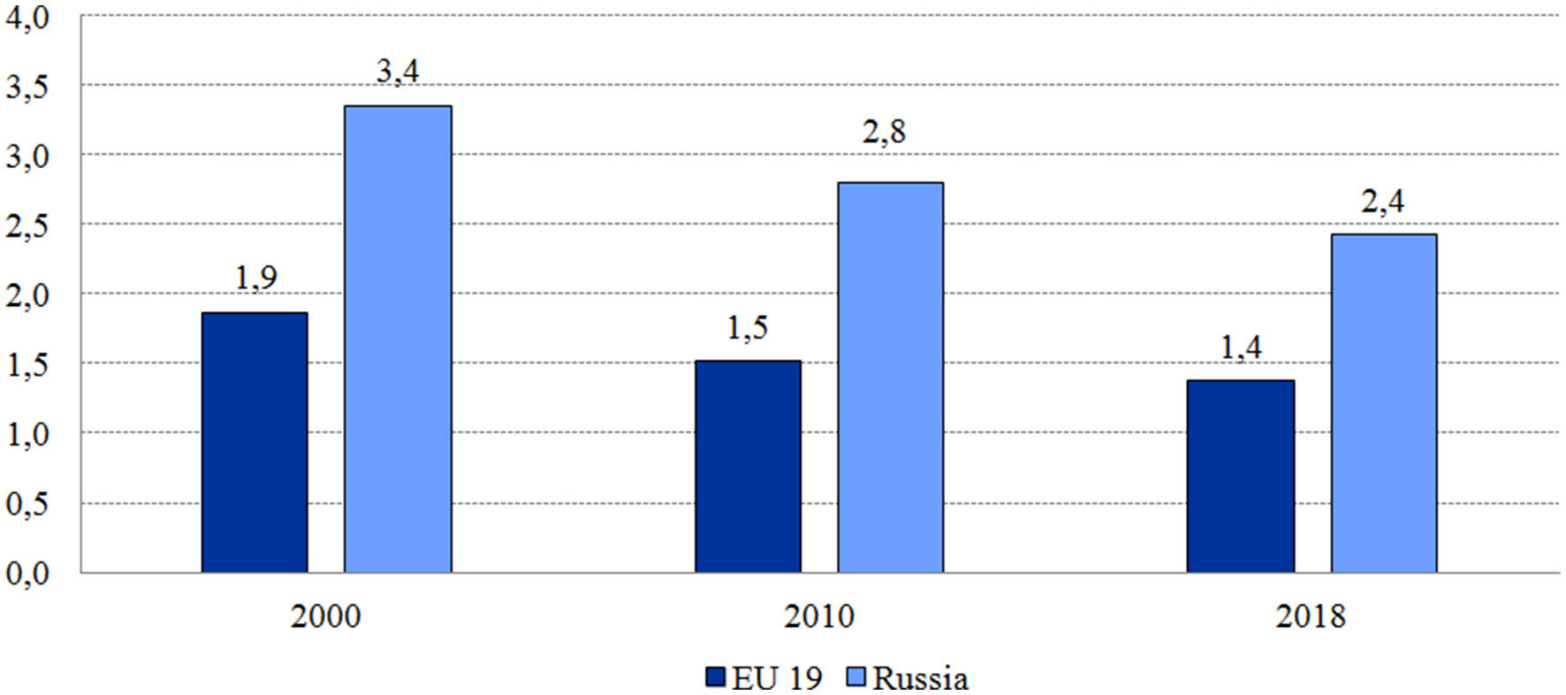
Number of hospital bed-days per person in Russia and the EU average. *Source*: (18).

The MHI model has become a catalyst for service delivery changes. Activity-based purchasing contributed to the shift of some inpatient care to outpatient settings and day care centers. This process has accelerated since 1999 under the annual federal utilization targets. The number of bed-days per capita decreased from 3.4 in 2000 to 2.4 in 2018—much faster than the EU average (Figure 3). Most of this decrease resulted from a substantial drop in the average length of hospital stay—from 15.5 to 10.7 days. The number of hospital admissions per 100 residents was stable (21.9 in 2000 and 22.4 in 2018) in contrast to the EU average over this period (18.4 in 2000 and 16.9 in 2018). Regional MHI funds encourage the deployment of day care centers by increasing their reimbursement rates. The share of patients treated in day wards in the total number of patients treated in hospitals increased from 7.6% in 2000 to 20.8% in 2018 (18).

### 4.4. Impact on the accessibility and quality of healthcare

In the budgetary system of healthcare finance, patients had almost no opportunity to choose providers. They were attached to specific state polyclinics in their place of residence, while hospital admission was strictly regulated by the pathways of patient flows in the region. An important advantage of the MHI in comparison to the budgetary system of health finance is that MHI allows patients to contract any provider irrespective of their ownership, including private providers. Providers of the “parallel” health system that have traditionally served only specific groups of residents (e.g., the staff of some ministries) can work currently under the MHI scheme. Patients can select a polyclinic (once a year) and a hospital (if referred by a polyclinic physician). Patients can also receive care outside their place of residence. The borders between territories are being erased by MHI.

The major mechanism of MHI's impact on the quality of care is quality control by health insurers. Health insurers check the claims of providers and identify “incomplete care,” that is, care violating clinical protocols as well as defects in medical recording (the latter is the most common). They can impose penalties on providers (they vary from 30 to 50% of the cost). Another form of quality control is the “thematic review” of clinical practice, that is, the identification of common mistakes in a selected clinical area.

Quality control by insurers has prompted serious discussion in the medical community. Health professionals often do not agree with the monitoring of their activities, which often comes down to checking that patients' medical records are filled out correctly. The ability of external experts to verify the performance of experienced clinicians has been questioned. With all these concerns, even simplified schemes of quality control allow insurers to mitigate the most visible manifestations of the poor qualifications of some doctors, negligence, and sometimes even clear violations of medical ethics (17).

## 5. Unsolved problems of MHI

### 5.1. Revenue collection

The current MHI system still lacks clear-cut rules for responding to the shortage of public funding. The budget of the MHI system is determined politically and is practically unconnected with the actual cost. The aforementioned targets of utilization and unit cost are based on budget estimates and are adjusted irrespective of the actual cost of services and healthcare needs. When a shortage of revenue is expected, these financial parameters are adjusted downward. This adjustment allows the government to formally balance MHI revenue with the government's commitments to free care. However, a real balance does not exist and the search for ways to reach it is irrelevant. Potential mechanisms of adjustment known internationally [longer waiting times targets for elective care, cuts in benefits packages, a rise in co-payment rates, encouraging voluntary health insurance, and higher requirements for the cost-effectiveness of new medical technologies (19)] are not used in Russia. They are replaced by the implicit rationing of healthcare without attempts to assess the potential outcomes (what can be cut and which cuts are impossible). For example, with the growing deficit of funding, existing federal targets of waiting times (for physician visits, diagnostic tests, and hospital admissions) are increasingly violated, but information on actual waiting times is not available. Uncertainty is a real problem. Patients understand that resources are limited, but do not understand why they are not told the actual waiting time. The uncertainty limits their opportunity to use alternative providers in other parts of the country or in the private sector (20).

When the actual shortage is not recognized by the government, then there is no clear claim for additional funding and there is no explicit cost containment policy. Flexible adjustment to the shortages gives way to the illusion of healthcare funding sustainability.

### 5.2. Pooling MHI revenue

Some medical services are still beyond the MHI system. Regional and municipal governments pay directly for public health, mental healthcare, cases of infectious diseases, AIDS, and some tertiary care. Investment expenditure is also covered by the government. The budgetary and MHI parts of the entire public health system currently function under separate regulations.

A special problem of pooling is that the bulk of investment costs are beyond the MHI system. These are covered by health authorities through budgetary subsidies. Decision-making on purchasing major medical equipment is not transparent. There is no link between the volume of provider activity and the allocation of funding. State-owned polyclinics and hospitals do not pay for major equipment as a result of its poor responsibility for their rational use. There are many examples of the underutilization of this equipment (13). This funding pattern also discriminates against private providers who are involved in MHI. They cover investment costs without government support, and their cost of services is usually higher than the current tariffs in the MHI system. Therefore, the level of private sector involvement in MHI remains low, and its services are provided mostly from out-of-pocket payments.

Internationally, the degree of pooling current and investment costs is much higher. Paris et al. (19) provided evidence that hospitals purchase major equipment jointly with governments of various levels in most European countries with MHI systems. In Germany and the Netherlands, hospital revenue is the major source of investment. In other words, hospitals earn resources for investment—their service reimbursement includes investment costs. The government does not lose the leverage of major investment regulation but recognizes the important role of providers in its funding.

### 5.3. Purchasing healthcare

**Figure 4:**
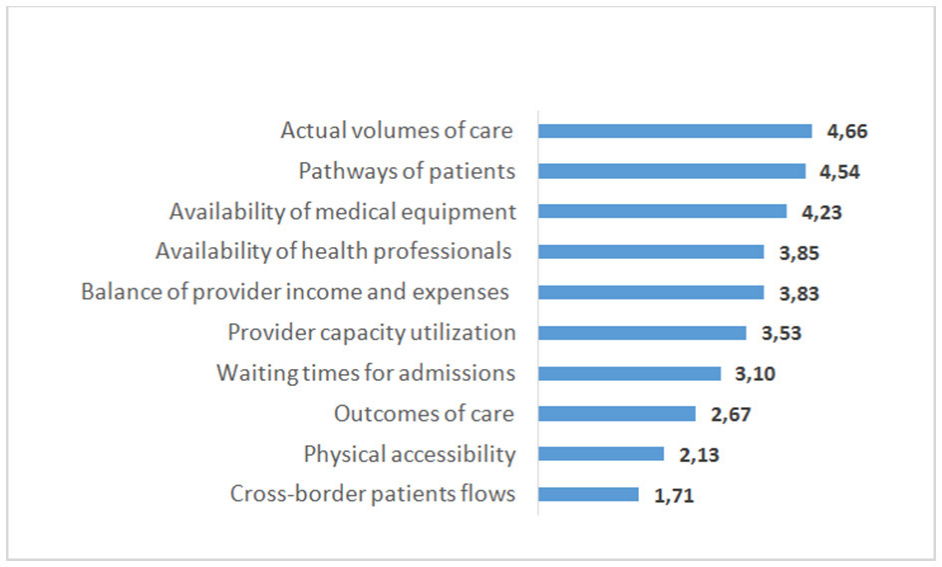
Average values of the criteria used by 86 regional MHI funds in Russia for the care volume allocation among medical care providers. *Note*: Respondents rated the value of each criterion on a 6-point scale from 0—not used, up to 5—high value. *Source*: The survey of regional MHI fund manager in 2019 (21).

The main problems of the current MHI model are concentrated in the purchasing of healthcare. The first problem is that care utilization planning and the allocation of volumes of care across providers are poorly focused on improving healthcare performance. A survey of the heads of 86 regional MHI funds conducted by HSE University in 2019 provides some insights into the specific criteria determining the allocation of volumes. They were estimated according to a 6-point scale with the average estimates given in Figure 4.

When distributing volumes of care across providers, the commission mostly takes into account last year's volumes of care and the prevailing patterns of patient movement in a multi-level system of service delivery (patient pathways). Next in importance is the availability of medical equipment and staff. Another important criterion is the need to ensure the financial stability of providers. The physical accessibility of providers and their performance characteristics are the least frequently used criteria.

There is practically no accounting for the quality of medical care, its complexity, or the development of new medical technologies. It is a common situation when a hospital develops a new medical intervention, treats the most complex patients, and has higher outcomes and shorter hospital stays compared with other hospitals in the region but receives the same planned volumes of inpatient care when the number of beds is equal (hence, the same funding). Contrary to many European countries, which increasingly account for the cost-effectiveness of alternative interventions, these important parameters are practically ignored in care purchasing in Russia. This approach hinders the development of new medical technologies.

Even these criteria are not transparent for health providers. We could not find information on the individual criteria on the websites of regional health authorities. This lack of information indicates that providers cannot compare their performance with their competitors or assess the fairness of the resource allocation.

The emerging private sector is contracted for the provision of services under MHI, but it accounts for only 5% of the entire volume of care. There are many barriers to its involvement. In general, 70% of survey respondents refer to the priority of state medical organizations in the allocation of volumes of care and 65% to the excessive and complicated reporting of private providers in the MHI system.

The same survey indicates that 70% of regional MHI leaders are happy with this pattern of care utilization planning and are not looking for ways to change it. The inertia of “simple solutions” is a strong factor in the (lack of) development of the system.

Related to this is the formal contracting between purchasers and providers. According to the legislation, health insurers select providers and determine the scope of services. However, the actual practice is based on a “typical contract” that consists of a standard set of provisions with references to the general regulatory requirements on service delivery. The scope of the negotiated contract parameters is very narrow. The volume of care is determined by the commission with no or little involvement of a health insurer as a contracting party. According to one respondent, the head of the MHI fund in a central Russian region, “*Health insurers in our region do not take an active part in the allocation of the volumes of medical care, while health providers often initiate changes in tariffs and in the allocation of resources*.”

Risk-sharing arrangements are unavailable in contracts. Therefore, the reimbursement of the overprovision of contracted volumes, as indicated earlier, is always a problem and is usually solved through the adjustment of contracted volumes. Risks of overprovision are shifted mostly to MHI funds.

The market pressure of selective contracting for providers is negligible. Providers may have some competitive advantages in terms of quality, but they must prove them in the commission where the negotiating procedure is focused on the volume of services and not the quality. While contracting providers, health insurers are not allowed to use methods of payment and pay-for-performance schemes differing from those determined by the regional MHI fund. Therefore, the capacity of health insurers to use their own instruments to encourage the provision of value-based care is unavailable. Also, medium- and long-term contracts with providers are not used, which hinders the realization of investment projects.

The prevailing pattern of contracting does not provide for multilateral arrangements to promote the integration of care. Contracts involving many providers to ensure their joint work on chronic disease management, continuity of care, and other integrative activities, which have become popular internationally (22), are unknown in Russia, although the need for them is growing. Attempts to integrate care are limited to mergers of providers without any real integrative activities under new contracting schemes (23).

Interviews with health leaders indicate a lack of interest in innovative practices of planning and contracting. According to the respondent from central Russia, “*We are too busy with the current problems, therefore do not have the opportunity to think about using alternative approaches to planning volumes of care*.”

Thus, contrary to declarations about negotiating volumes of care between purchasers and providers, the pattern of resource allocation has more resemblance to the Soviet style of directive planning.

## 6. Discussion

The analysis indicates that the collection function in the Russian MHI system is based on state regulations with the separation of powers between federal and regional governments, while the revenue of MHI funds is separated from the general budgetary revenue.

The function and rules of pooling are based on state regulations, including the accumulation of contributions in the federal MHI fund with their further allocation to the lower levels of accumulation—in regional MHI funds and health insurers. The governance of funds has elements of societal regulation. The implementation of pooling in the MHI system is separated from the budgetary system. Healthcare purchasing is based mainly on state regulations with the minimum use of societal and market regulation. The major role is played by federal and regional governments. Thus, the current MHI system maintains elements determined by its initial design of building a competitive model of MHI with the major role of the market regulation—multiple insurers and their interaction with regional MHI funds and health providers. While the actual performance of this system is completely regulated by the state.

The dominant role of state regulation distinguishes the Russian model from MHI models in Western Europe, where societal regulation plays a major role (24, 25) and makes it closer to the MHI models in Central and Eastern European countries—Czechia, Hungary, Poland, Slovakia, and Estonia (26). However, unlike the countries of Central and Eastern Europe, which have MHI systems of this type, the Russian system is not totally separated from the state budgetary system. It is separated from the budgetary system in revenue collection and pooling, while it is not separated in purchasing. The latter has a hybrid regulation that combines state regulation used in the budgetary system with regulations (state, societal, and market) used in the health insurance systems.

The introduction of the competitive model of MHI in Russia has been limited to multiple private insurers. The institutions to promote their competition have not been built.

Health insurers may look like the vestiges of the market model of MHI, but in the specific conditions of the Russian healthcare governance, they perform the important function of deterring violations of patients' rights by health providers and officials. The liquidation of insurers will most likely make patients totally dependent on health providers and officials.

Were other expectations of the MHI model met? The Russian MHI system has improved the structure of service delivery and promoted patient choice and the cross-border movement of financial resources and patients. However, the hybrid nature of health purchasing in the Russian public administration system limits its impact on the performance of the health system.

The actors of the MHI system do not have sufficient motivation to improve the performance of the healthcare system. MHI funds are state-owned institutions that report to the government. Their priority is the fulfillment of tasks formulated by higher levels of government and ensuring the stable operation of state medical organizations. The most efficient use of resources is of little relevance to MHI funds.

Health insurers are primarily required to ensure financial support for the stable operation of health providers and to protect the rights of patients. Insurers have practically no opportunities and incentives to select the best providers for their clients or improve the efficiency of using MHI financial resources. However, the rules of MHI create some economic motivation for health insurers to monitor the quality of healthcare and the appropriateness of providers' bills. This is based on financial penalties imposed on providers and the right to keep a certain percentage of these penalties.

Health providers are interested in maximizing the revenue received from the MHI system. However, there is no strong economic pressure from health insurers, other providers, or patients, which may force them to optimize their costs and improve the quality of care.

People cannot choose insurers based on their promises to monitor the quality of care. This monitoring does not provide the information required by individuals on where and how to receive value-based care. This lack of information limits the ability of citizens to exert competitive pressure on insurers and providers, which would stimulate them to improve their work.

Major components of the strategic purchasing conceptual framework (promoted by the European office of the WHO) are not widely used in Russia. Empowering citizens is in its infancy since there are no specific policies that incorporate citizens' views into purchasing decisions. Incorporating cost-effective contracting has been discussed, but there are no strong incentives to implement it. The government is developing some activities to strengthen its stewardship (training health managers and outsourcing some services to the private sector), but they are not enough to improve the use of cost-effective contracting. Similar to many other European countries (27), there is little evidence in Russia of purchasing health insurance being strategic according to any of the established definitions (21).

The dilemmas in the further development of the Russian MHI system are whether (a) replacing the remaining elements of the societal and market regulation with state regulation, eliminating the separation of the MHI system, and integrating it into the budgetary system or (b) maintaining its separation from the budgetary system and attempting to strengthen the societal and the market mechanisms of regulation, including strengthening the role of health insurers, are helpful for the development.

The first alternative is very likely in the current political and economic situation. However, with this choice, the problems discussed earlier will persist and be more difficult to address. Dismantling the MHI system would provide very small administrative savings, but it would require building new legislative and operational mechanisms in the budgetary system for the purchaser–provider split, including contracting and changing the functions of health authorities of all levels.

The second alternative would create the conditions for sustainable progress in the performance of the MHI system and its more substantial contribution to strengthening the healthcare system. This would require a consistent state policy of developing regulatory mechanisms that are alternative to the administrative governance of healthcare.

## 7. Conclusion

During the transition from the planned Soviet economy to a market economy, an attempt was made in Russia to replace the budgetary model of health finance with the MHI model. The original intention was to introduce a competitive model, but this has not been realized; the resulting model is a hybrid one with three main characteristics.

First, MHI has not completely replaced the system of budgetary funding—some healthcare provisions and investment costs are still financed with the use of budgetary model mechanisms.

Second, the MHI system is not completely separated from the system of budgetary funding. It is separated in the collection and pooling of funds, while the purchase of healthcare combines elements of both models.

Third, the current model is a unique combination of state regulation and societal and market regulation. The latter are the rudiments of the initial design of the model that has not been fully realized.

The initial expectations have been only partially met. The MHI model has contributed to more stable health funding, to its geographical equalization, and to service delivery restructuring. However, the finance functions have many serious unsolved problems, which require a change in the design of the model.

The analysis of the Russian MHI system allows us to formulate the following lessons for countries considering the possibility of replacing their budgetary health financing systems with the MHI systems.

The main lesson is that the MHI system regulated only by the state enhances the effectiveness of the pooling (and distribution) function but creates obstacles to the purchasing function. Developing the mechanisms of strategic purchasing is a serious problem.

A competitive MHI model is not automatically ensured by having multiple health insurers and contracting health providers but requires a diversity of benefit packages, rates of insurance premiums, selective contracting, and schemes for service reimbursement. Developing these institutions require long-term efforts by health policymakers.

A clear understanding is needed that, with weak democratic institutions, the government will most likely cope with the unsolved problems of the MHI system by replacing or supplementing the institutions of societal and market regulations with state regulations. Such a policy may lead to some positive outcomes for healthcare performance in the short term, but their impact in the longer term remains undetermined. A major lesson is that market and societal regulation are poorly compatible with weak democratic institutions.

## Data availability statement

Publicly available datasets were analyzed in this study. This data can be found at: https://rosstat.gov.ru/.

## Author contributions

Both authors listed have made a substantial, direct, and intellectual contribution to the work and approved it for publication.
